# A Rare Case of Concurrent Chilaiditi Syndrome and Acute Appendicitis: An Uncommon Diagnostic Dilemma With Literature Review

**DOI:** 10.7759/cureus.81137

**Published:** 2025-03-25

**Authors:** Rafique Umer Harvitkar, Ioannis Hannadjas, Jiahui Ren, Ahmed Abdelgany, Abdul Malik Magsi

**Affiliations:** 1 General Surgery, Royal Sussex County Hospital, Brighton, GBR

**Keywords:** appendicitis, chilaiditi syndrome, gastrointestinal anomaly, laparoscopic appendicectomy, volvulus

## Abstract

Chilaiditi syndrome, characterized by the interposition of the colon between the diaphragm and the liver, is a rare anatomical variant often discovered incidentally during imaging for unrelated conditions. While typically asymptomatic, it can occasionally lead to gastrointestinal symptoms or complications such as bowel obstruction, volvulus, or even appendicitis, an exceptionally rare occurrence. We report the case of a 60-year-old female diagnosed with Chilaiditi syndrome who developed acute appendicitis. She underwent a successful laparoscopic appendicectomy with an uncomplicated postoperative recovery. This case underscores the diagnostic challenges of Chilaiditi syndrome, particularly when coexisting with acute abdominal conditions, and highlights the importance of advanced imaging, such as CT, in atypical presentations.

## Introduction

Chilaiditi syndrome is a rare radiological finding involving the interposition of a portion of the colon, most commonly the transverse or sigmoid colon, between the diaphragm and the liver. First described by the Greek radiologist Demetrius Chilaiditi in 1910, it generally presents as an incidental discovery during imaging for unrelated conditions [1]. While many individuals with this anatomical variant remain asymptomatic, some may experience gastrointestinal symptoms including abdominal pain, bloating, nausea, vomiting, and alterations in bowel habits. The estimated incidence of Chilaiditi syndrome in the general population ranges from 0.025% to 0.28%, with a higher prevalence in individuals aged 50 years and older [2,3].

Although Chilaiditi syndrome itself is typically benign, it can be associated with complications such as bowel obstruction, volvulus, and, on rare occasions, appendicitis. The precise pathophysiology remains unclear but is thought to involve anatomical anomalies of the colon, increased intra-abdominal pressure, or developmental variations preventing the diaphragm from fully fusing with the liver [3-6].

Acute appendicitis is one of the most common causes of acute abdominal pain, with a lifetime risk of approximately 7% to 8% in the general population [7-10]. The coexistence of appendicitis and Chilaiditi syndrome is exceptionally uncommon, with only limited reports in the literature. The diagnostic challenge arises from overlapping symptoms with other conditions, such as acute cholecystitis or gastritis, particularly when right upper quadrant (RUQ) pain predominates. This case report highlights the diagnostic challenges posed by the rare coexistence of Chilaiditi syndrome and acute appendicitis, emphasizing the importance of accurate imaging and a tailored management approach

## Case presentation

A 60-year-old female with a medical history of right mastectomy for breast cancer presented with a two-day history of RUQ abdominal pain, nausea, vomiting, and a recorded fever of 38°C. She reported recurrent episodes of epigastric discomfort in the past, previously treated as gastritis, without any prior history of bowel obstruction or significant gastrointestinal issues. She denied recent trauma and had no history of abdominal surgery aside from her mastectomy.

On examination, she was febrile, with notable tenderness over the RUQ and mild abdominal distension. A positive Murphy’s sign was elicited. Laboratory tests revealed leukocytosis with a white blood cell count of 16x109/L and an elevated C-reactive protein level of 82 mg/L. Liver function tests remained within normal parameters, and renal function was stable (Table 1). Given the clinical presentation, an initial diagnosis of acute cholecystitis was considered. To confirm the diagnosis and explore other potential causes, a contrast-enhanced CT scan of the abdomen and pelvis was performed.

**Table 1:** Blood test results

Tests	Result	Range
Serum C-reactive protein	82	0 - 5 mg/L
Haemoglobin	111	115 - 165 g/L
White blood cells	16	4 – 10 x10^9^/L
Serum total bilirubin	18	0 – 21 umol/L
Serum urea	4.0	2.8 - 8.1 mmol/L
Serum creatinine	66	45 – 84 umol/L

**Figure 1:**
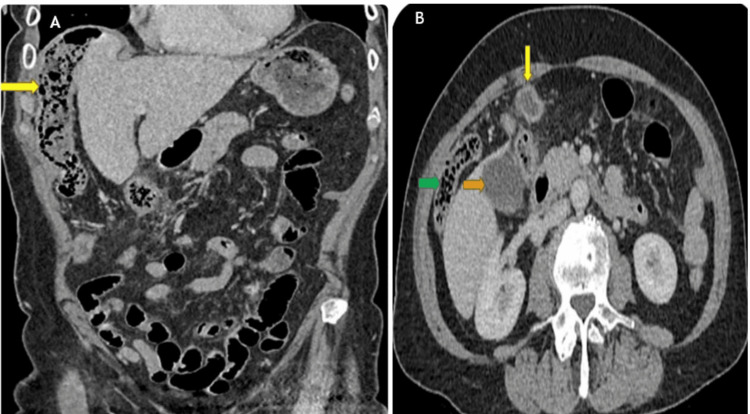
CT of the abdomen and pelvis A: Coronal view shows Chilaiditi syndrome with the colon interposed between the diaphragm and liver (yellow arrow); B: Sagittal view shows the right colon trapped between the diaphragm and liver (green arrow), gallbladder (orange arrow), and appendix (yellow arrow)

The CT imaging revealed a displaced caecum and appendix in the RUQ, with the appendix appearing dilated and thickened, surrounded by periappendiceal inflammatory changes (Figure 1). No evidence of perforation or drainable collections was noted. The anatomical displacement of the caecum and appendix between the diaphragm and liver was consistent with Chilaiditi syndrome, which had not been previously identified in this patient. The clinical diagnosis was revised to Chilaiditi syndrome with complicated acute appendicitis.

**Figure 2:**
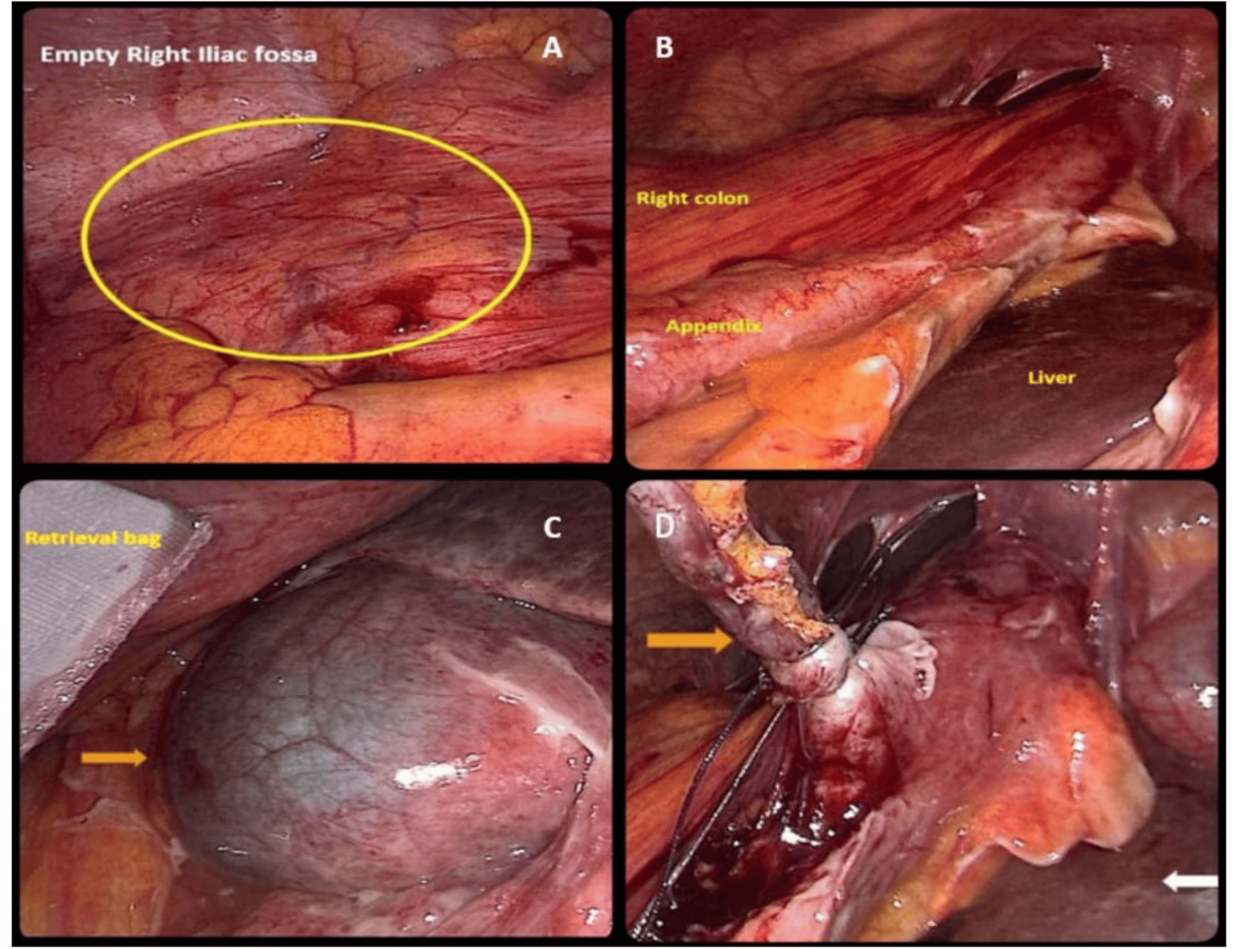
Laparoscopic findings A: Empty right iliac fossa (encircled area); B: Inflamed appendix adjacent to the right colon and liver; C: Gallbladder (orange arrow) and retrieval bag containing the appendix; D: Orange arrow points at the appendix secured with an endoloop and the white arrow points at the liver

The patient was promptly scheduled for a laparoscopic appendicectomy. Intraoperatively, the transverse colon and right colon were found interposed between the abdominal wall and the liver, confirming the diagnosis (Figure 2). The appendix was located over the right lobe of the liver and was grossly inflamed with signs suggestive of impending perforation at the tip. The gallbladder appeared normal, although localized adhesions and purulent fluid were observed around it. There was no evidence of bowel obstruction, and the large bowel was left untouched to avoid unnecessary manipulation. The appendicectomy was completed without incident, and the peritoneal cavity was irrigated with saline to clear inflammatory debris.

Postoperatively, the patient received intravenous antibiotics and demonstrated an uneventful recovery. She was discharged on the second postoperative day with a five-day course of oral antibiotics and remained asymptomatic at her two-week follow-up visit.

## Discussion

Chilaiditi syndrome is an infrequent clinical and radiological finding that presents significant diagnostic challenges when associated with other intra-abdominal pathologies. Its incidence, although variable, tends to increase with age, chronic constipation, liver cirrhosis, and conditions that elevate intra-abdominal pressure. Most patients are asymptomatic, but when symptomatic, the presentation can mimic other abdominal conditions, including acute cholecystitis, pancreatitis, and myocardial infarction [4-7].

The coexistence of Chilaiditi syndrome with acute appendicitis is notably rare. The few cases documented in the literature describe patients presenting with atypical pain patterns, often in the RUQ rather than the classic right lower quadrant (RLQ) distribution associated with appendicitis. This positional anomaly of the appendix, as observed in our patient, can delay diagnosis and treatment if not promptly identified through advanced imaging techniques such as CT scans. High clinical suspicion remains essential, particularly when initial investigations suggest alternative diagnoses [7-10].

The pathophysiological mechanisms underlying Chilaiditi syndrome involve a combination of colonic redundancy, elongated mesocolon, lax hepatic suspensory ligaments, and diaphragmatic anomalies. Secondary factors, including chronic lung disease, obesity, and prior surgeries, can further predispose patients to this anatomical variation. Although our patient had no clear predisposing factors aside from age, her history of chronic epigastric discomfort may have been an underappreciated symptom of intermittent colonic displacement [9-11].

Existing literature, including case reports by Lens et al., Augustin et al., Devecki et al., and Rogelio et al. emphasizes the importance of early imaging and surgical management to prevent adverse outcomes [ 9-12]. Our case supports these findings, underscoring that prompt recognition and intervention can result in favorable patient outcomes without added morbidity.

Management of Chilaiditi syndrome is typically conservative unless complications arise. In cases where appendicitis is present, surgical intervention becomes necessary. Laparoscopic appendicectomy is the preferred approach due to its minimally invasive nature and enhanced visualization, which is especially advantageous in patients with atypical anatomy. Careful intraoperative handling is crucial to avoid iatrogenic injuries to the displaced bowel segments. In our case, the decision to refrain from manipulating the large bowel minimized the risk of volvulus or obstruction, which has been documented in other reports.

## Conclusions

Chilaiditi syndrome is an uncommon anatomical variant that can present with non-specific gastrointestinal symptoms and may complicate the diagnosis of acute abdominal conditions such as appendicitis. This case highlights the importance of considering Chilaiditi syndrome in the differential diagnosis of RUQ abdominal pain, particularly when imaging reveals unusual anatomical relationships. Advanced imaging modalities and a high index of clinical suspicion are pivotal for timely diagnosis. In cases complicated by appendicitis, early laparoscopic appendicectomy remains an effective treatment, ensuring a favourable outcome when performed without delay.
